# The Role of Pharmacists in Counteracting Vaccine Hesitancy: Effectiveness of the 2019 Carnia Project in Improving Adherence to Influenza Vaccination among Target Population

**DOI:** 10.3390/vaccines12030331

**Published:** 2024-03-20

**Authors:** Gloria Longobardi, Laura Brunelli, Benedetta Piciocchi, Andrea Morsanutto, Andrea Iob, Flavio Schiava, Claudio Luigi Pancino, Luca Degrassi, Giuseppe Tonutti, Silvio Brusaferro, Luca Arnoldo

**Affiliations:** 1 Dipartimento di Medicina, Università degli Studi di Udine, 33100 Udine, Italy; 2 SOC Accreditamento, Qualità e Rischio Clinico, Azienda Sanitaria Universitaria Friuli Centrale, 33100 Udine, Italy; 3 Assistenza Farmaceutica Territoriale, Azienda Sanitaria Universitaria Friuli Centrale, 33100 Udine, Italy; 4 Dipartimento di Prevenzione, Azienda Sanitaria Universitaria Friuli Centrale, 33100 Udine, Italy; 5 Federfarma Friuli Venezia Giulia, 33100 Udine, Italy; 6 Azienda Regionale di Coordinamento per la Salute, 33100 Udine, Italy

**Keywords:** vaccine hesitancy, pharmacy, counseling, influenza vaccination

## Abstract

Vaccine hesitancy has been included among the top ten threats to global health by the World Health Organization. Pharmacists can play a pivotal role in removing the individual barrier to vaccination, because of the relationship of trust they have with citizens and their ease of access. The aim of this study was to examine the impact of a pharmacy-based intervention to support the 2019 influenza vaccination campaign conducted in the Carnia district through one-to-one counseling. We analyzed data collected by pharmacists between 22 October 2019 and 20 January 2020, and trends in vaccination adherence in the context of the Local Health Authority and the entire province of Udine since 2016. The results showed that 77.2% of people who had not received an influenza vaccination in the previous year changed their minds about vaccination after receiving counseling. The pharmacy-based intervention improved influenza vaccination adherence in the target district (+13.4%), even when compared to the neighboring district of Gemona or considering the data in the broader local and provincial context, and this effect was particularly pronounced among those aged 65 to 74 years (*p* < 0.01). Considering these findings, pharmacies should be more effectively involved in the provision of public health services aimed at improving accessibility, timeliness, and equity.

## 1. Introduction

Vaccine hesitancy has been defined by the World Health Organization’s Strategic Advisory Group of Experts (SAGE, WHO) as a delay in accepting or refusing immunization despite the availability of immunization services [1]. The scale of this phenomenon is significant, so even in the pre-pandemic period, it was estimated that the proportion of hesitant parents could reach 16% of the total Italian population [2]. For this reason, the WHO has included vaccine hesitancy among the top ten threats to global health [3], and in recent years the aim has been to reduce it in every possible way. Because it is a complex and context-specific problem that varies with temporal, spatial, and vaccine-specific variables [1], the European Center for Disease Prevention and Control (ECDC) recently revised the earlier 3C model [4] and proposed a new version that includes 5Cs: Confidence, Constraints, Complacency, Calculation, and Collective responsibility [4,5,6,7,8].

In addressing this complex public health issue, pharmacists can play a pivotal role when it comes to removing the individual obstacles to vaccination. In fact, pharmacists are often the first point of contact for patients and the public, both because of the bond of trust they have with citizens and because they are easy to access, which results from the wide distribution of pharmacies in the area, the availability of longer opening hours, the lack of need for appointments, and their proximity to healthcare facilities [9]. Recognizing these benefits, they can work for public health by helping to identify people with special risk factors or unhealthy lifestyles, and they can provide evidence-based education and advice, dispel common misconceptions about vaccination, and potentially prevent future outbreaks of vaccine-preventable diseases, as already suggested [10,11]. Indeed, communication is a fundamental tool for interacting with citizens: when poor or inadequate, as in the case of information overload [12], it can negatively impact immunization adherence, undermine the acceptability of the proposed immunization schedule, and compromise health outcomes for an entire population. Programs that implement pharmacy-based interventions to support immunization help increase the effectiveness of immunization campaigns, particularly with regard to that part of the population which, for various reasons, is not well or frequently reached by general practitioners or primary care services [11]. With this in mind, countries have explored different ways of involving pharmacists in their campaigns. For example, in Catalonia, Spain, the introduction in 2017 of a new influenza surveillance system made it possible to achieve high levels of anti-flu vaccination coverage through the involvement of a network of sentinel community pharmacies that not only carried out vaccination education and promotion activities but provided important epidemiological surveillance data [13]. In Ireland, where pharmacists have been shown to be the healthcare professionals most in contact with the public, the Pharmaceutical Society of Ireland (PSI) has developed a modular training system to equip pharmacists with the skills and knowledge needed to safely administer drugs and vaccines [11]. In Canada, allowing pharmacists to inject influenza vaccines has been shown in recent years to increase adherence to the influenza vaccination campaign [14,15]. Although Italian pharmacists were not directly involved in vaccination campaigns in the pre-pandemic era, they could already play a key role in informing and advising consumers and promoting public health. In particular, Law No. 119/2007 reaffirmed the central role of pharmacists in promoting prophylactic vaccination by explicitly stating in Article 2 that the Italian Ministry of Health can count on the collaboration of pharmacists as well as general practitioners and pediatricians [16]. Moreover, the Italian Code of Conduct for Pharmacists (Codice Deontologico del Farmacista), last updated in 2018, reaffirms the commitment of the pharmacy profession to participate in prevention and health education campaigns promoted by the competent authorities [17]. The Italian Federfarma, one of the main professional associations of pharmacists, has also advocated activities to support health services for prevention and health promotion programs [18,19,20].

**Figure 1:**
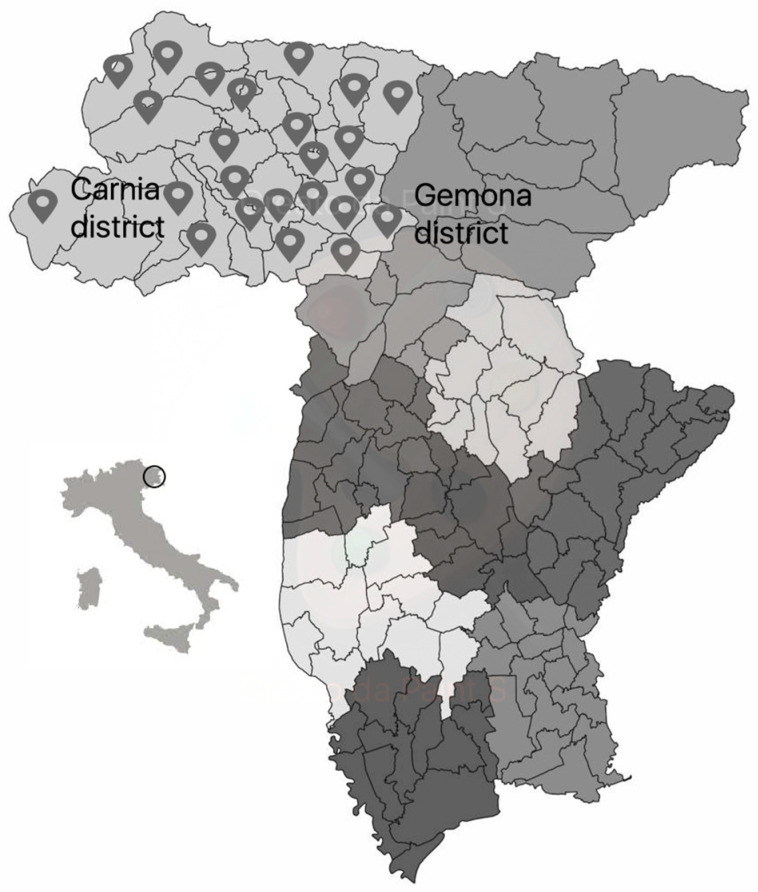
Health districts of the province of Udine (northeastern Italy), with the geographical localization of participating pharmacies within Carnia district identified by a pinpoint.

In 2019, a vaccination counseling project to support the influenza vaccination campaign was launched in the Italian province of Udine (Friuli Venezia Giulia Region—FVG), based on a specific agreement between the Local Health Authority (LHA, Azienda per l’Assistenza Sanitaria No.3, AAS-3) and Federfarma FVG. The project was implemented in the Carnia district, a mountainous area in the northern part of the province of Udine (as illustrated in Figure 1) characterized by logistical difficulties in accessing vaccination centers and health services in general, which has led to low influenza vaccination rates in recent years [21]. Although it has a very large area (approximately 1222 square kilometers) with a low population density (37,215 inhabitants in 2019), it was considered an ideal place to carry out the project because pharmacists play an important role in the daily lives of these communities.

The primary aim of this study was to examine the impact of the vaccination counseling project implemented by pharmacists in the Carnia district, by evaluating data on the number of users who participated in the 2019 seasonal influenza vaccination campaign after receiving counseling at local pharmacies participating in the project. A secondary aim was to compare the percentage of people vaccinated in the Carnia district during the 2019–2020 flu season with that in another district in the same LHA where the project was not implemented. Another aim was to compare the percentage of vaccinated people in the contexts of the LHA and the province of Udine and analyze the trend since 2016.

## 2. Materials and Methods

The project involved 44 pharmacists, at least one for each of the 24 pharmacies in the Carnia district, who received face-to-face training on influenza vaccination by professionals from the Department of Prevention to promote vaccination in one-to-one counseling sessions and eliminate any doubts about the effectiveness and safety of the vaccines. The topics discussed during the course are listed as Supplementary Materials to this paper (Supplementary Material S1). The target population for counseling activities consisted of persons potentially eligible for influenza vaccination according to current national and regional recommendations [22]: persons who were 65 years of age or older, or had at least one risk factor regardless of age (immunocompromised or with chronic diseases).

This study was designed as a retrospective observational study of data collected during the 2019–2020 influenza season as part of the project conducted with Carnia district pharmacies. We analyzed data collected by pharmacists between 22 October 2019 and 20 January 2020, through a brief interview in the Italian language to determine if individuals had been vaccinated in the past year and if they were willing to get vaccinated against influenza during the current flu season. The draft of the interview is published as Supplementary Materials (Supplementary Material S2). If the participant already intended to get vaccinated, the interview ended with a supportive message from the pharmacist. If doubt, concern, or refusal was reported, the pharmacist asked about the reasons for the decision not to get vaccinated and offered a counseling intervention to the hesitant person. Additional data about the presence of risk factors (immunocompromised status, presence of chronic disease) were also collected for all participants. Participants were informed of the goal and methods of the project and gave their consent to participate. On the basis of a framework agreement signed between the LHA, Federfarma FVG, and the federation of socio-pharmaceutical companies and services, the pharmacies participating in the project received a financial incentive based on the number of counseling sessions performed (EUR 1.00 + VAT). After the counseling, if the user who had not been vaccinated in the previous year decided to get vaccinated, the pharmacy received an additional reward share (EUR 5.00 + VAT).

We also analyzed trends in vaccination adherence in the context of the LHA and the entire Udine province since 2016 by obtaining data from aggregated regional vaccination records. The study protocol was approved by the Institutional Review Board of the University of Udine, Italy.

We included only individuals aged 65 years and older because age is the only risk factor for influenza vaccination that can be retrospectively obtained from the regional database without bias and allows comparisons with previous years and other districts. In addition, for the specific analysis of behavior change among participants, we only included individuals who were already 66 years old during the 2019–20 vaccination campaign, to ensure that they all met the criteria for influenza vaccination. For the analysis of the evolution of vaccination adherence, data were filtered to obtain all patients who turned 65 years old at the end of the year studied. To analyze the presence of risk factors as a potential confounder, we subdivided the study population into two groups, considering people with and without other risk factors as registered by pharmacists. To understand the impact of the project, we compared the influenza vaccination adherence of the Carnia district with that of the neighboring district of Gemona, the LHA, and the entire province of Udine considering data from 2016 to 2019. Actions and strategies implemented in these four progressively expanded settings and compared with the analyses are listed in the Supplementary Materials (Supplementary Material S3). To determine whether the effectiveness of the intervention varied with the age of the target population, we divided the over-65 population into three subgroups as follows: 65–74 years, 75–84 years, and over 85 years. Statistical analyses were performed using the McNemar test for evaluating adherence to flu vaccination before and after counseling, as well as the Chi-square test and Chi-square test for trend, assuming as statistically significant a *p*-value < 0.05. Analyses were performed using the software IBM SPSS 20 Statistics. 

## 3. Results

The number of participants aged ≥65 years who attended the counseling promoted by local pharmacists amounted to 2109, against the 10,772 over 65 years of age residing in the Carnia district in 2019. More specifically, they included 1201 women (56.9%) and 908 men (43.1%). The mean age was 75 years (SD 7), while the most common age was 73 years (range 65–99 years). In total, 622 of the participants over 65 years of age had at least one comorbidity (29.5%) reported by the pharmacists: most of them were chronic diseases of the cardiovascular system (68.0%) and diabetes mellitus (17.0%).

Counseling activity took place mainly in the first two months of the flu vaccination campaign: October (687; 32.6%) and November (1081; 51.3%), while only 316 (15.0%) and 25 (1.2%) participants received counseling in December and January, respectively. Considering those who received counseling and were already eligible for influenza vaccination in the previous year, the total number of persons aged 65 years or older included in the behavior change analysis was 2020. The behaviors of the entire sample and its subgroups during the two subsequent influenza seasons are shown in Table 1. Reasons given for not vaccinating against influenza in the previous year included the belief that they would never get sick (43.0%), that vaccines are dangerous (11.1%), that they were unaware of the possibility of getting vaccinated against influenza (8.1%), and that vaccines were not effective (5.2%). The rest gave other answers that do not fall under the above options (32.6%). After counseling, a total of 1569 participants (77.7%) got vaccinated, of whom 591 belonged to the group that had not been vaccinated against influenza in the previous year. Statistically significant changes in behavior were found when considering the entire group of participants, and even when subdivided by the presence of additional risk factors.

**Figure 2:**
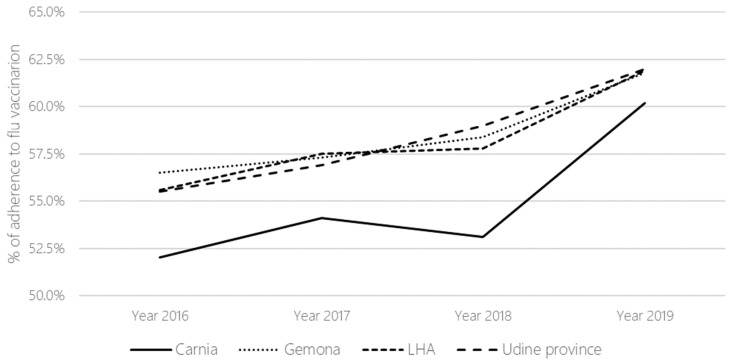
Influenza vaccination rates among people over 65 years of age from 2016 to 2019 at the district, LHA, and provincial levels.

Looking at vaccination adherence data since 2016, there was already a trend of significant increase in influenza vaccination adherence at the provincial and LHA levels, (+5.1% and +7.0%, respectively, in 2018–19), with a greater improvement noted for the Carnia district (+13.4%) in the year of the pharmacy-based intervention. Immunization rates from 2016 to 2019 among persons 65 years and older are shown in Table 2 and visually depicted in Figure 2.

When looking at subgroups, we found that in the 2019–2020 season, the people of Udine province aged 65 to 74 years reported the greatest improvement in vaccination adherence (+8.2%, *p* < 0.01 from 32,893/67,447 in 2018–2019 to 35,710/67,648), which was more than 1.5 times higher than the average implementation in the over 65 population (5.1%; from 82,086/139,230 in 2018–2019 to 87,256/140,809 in 2019–2020). In particular, the same subgroup in the Carnia district showed the highest and most significant increases (*p* <0.01): +18.6% (from 2270/5299 in 2018–2019 to 2690/5293 in 2019–2020).

The other subgroups of Carnia district, compared with those of Udine province, showed, respectively, +10.2% among those aged 75 to 84 (from 2327/3819 in 2018 to 2019 to 2614/3892 in 2019 to 2020), almost three times the average improvement in the same age group (3.5%, 33,198/50,452 in 2018–2019 to 35,083/51,526 in 2019–2020) and +8.6% among those aged 85 and older (from 1088/1585 in 2018 to 2019 to 1183/1587 in 2019 to 2020), almost six times the average increase in the same age group (1.5%, from 15,995/21,331 in 2018–2019 to 16,463/21,635 in 2019–2020).

## 4. Discussion

The aim of this study was to examine the impact of a pharmacy-based intervention to support the influenza 2019 vaccination campaign conducted in the Carnia district through one-to-one counseling. The results showed that 591 people aged 65 years or older who had not received the influenza vaccine in the previous year changed their minds about vaccination after receiving counseling. The main reasons for not getting vaccinated discussed during counseling included the belief that they would never get sick, the danger of vaccines, not knowing about the possibility of getting vaccinated against influenza, and the belief that vaccines are ineffective. The pharmacy-based intervention improved influenza vaccination adherence in the target district, even when compared to the neighboring district of Gemona or when the data are considered in the broader LHA and provincial context, and this effect was particularly pronounced among 65- to 74-year-olds.

Even though there had already been an increasing trend in vaccination adherence in the whole LHA and in the province of Udine since 2016, when comparing the increase in vaccination compliance between the districts of Carnia and Gemona since 2018, we found that the performance of the former district was better. This is true despite the similar geographic context, low population density, and the fact that the two districts belong to the same LHA. Therefore, the increase in vaccination adherence observed in the Carnia district between the 2018–19 and 2019–2020 campaigns may be due to the pharmacy-based intervention. Nonetheless, the effectiveness of this project may have benefited from the provision of an economic incentive to pharmacists, the long-term sustainability of which will need to be carefully evaluated in order to transition this evidence-based practice into a standardized process. However, there may have been other factors that contributed to the increase in vaccination rates in the Carnia district during the 2019–20 campaign. For example, the fact that the vaccine offered to persons 65 years of age and older was, for the first time, a quadrivalent adjuvanted vaccine, rather than the trivalent vaccine previously offered, and that the availability of doses in the Friuli Venezia Giulia region was greater (+50,000 doses compared with the previous year). The finding that more than 80% of people sought counseling by the end of November may be related to the recommendation of the health authority to vaccinate the population early that year, as the peak of influenza was expected in early December [23]. On the other hand, our study showed that some people who were vaccinated during the 2018–19 campaign (276 people aged 65 years or older) decided not to get vaccinated the following year. Analyzing the possible reasons for this decision, we found that influenza in 2018–19 actually affected the pediatric age group the most and spread mainly in central and southern Italy [24]. This particular distribution may have led older people in the northern part of the country, where the FVG region is located, to believe that they would not get the disease or that vaccination was not necessary. Moreover, some may believe that vaccination was ineffective because their vaccination anticipated the peak of the epidemic too much and so they became ill with influenza because of reduced vaccine effectiveness. After two seasons in which the epidemic peak occurred early (about four weeks in advance), it returned in 2018–19 to its usual timing between late January and early February [24]. In order to prevent this phenomenon from consolidating as a trend, it is important to underline the importance of carrying out positive reinforcement on subjects who had already been vaccinated in previous years.

Vaccine hesitancy remains a public health problem that needs to be addressed to improve vaccination coverage and health outcomes in the population. Therefore, strategies to address vaccine refusal should be carefully tailored to the target population, their reasons for hesitating, and the specific context [25]. One such strategy that is increasingly being used worldwide is to involve pharmacists in immunization campaigns to leverage their role in the communities in which they work, which is a powerful mix of expertise and close contact with the population, sometimes enriched by a potential liaison role between the patient and the referring specialist [15,26]. Indeed, in small communities, it may be easier to establish a trusting relationship with people because pharmacists know patients and their families and can better contextualize problems and potential solutions to specific issues [27]. As outlined in the literature, the role of pharmacies has changed profoundly in recent years, moving from simply preparing and distributing medications to an increasingly central role in patient management. This change was accelerated by the COVID-19 pandemic, in which pharmacists were at the forefront of infection prevention and control programs by providing swabs, distributing medical supplies, caring for patients, and, in some cases, administering vaccines [28,29], making more evident the variety of services pharmacies provide to the general population [29]. The provision of preventive services by pharmacies has been shown to be particularly effective in areas where the population has less ready access to other healthcare providers because of geographic characteristics and the existing distribution of services [15,30,31,32]. In addition, the new range of services offered by pharmacies that were traditionally provided only by hospitals and general practitioners can be seen as a key element for ensuring equitable and timely health care [29,30,33]. In our study, we focused on the potential impact of influenza vaccination counseling on vaccination adherence, but the role of pharmacists as public health providers has been successfully tested in many other settings, including immunization against diseases other than influenza [29,34,35], counseling and provision of dietary supplements [36] and generic substitution [37], HIV rapid testing sites [26], and checkups for vascular diseases [29]. In agreement with the reports of other authors [26,34,38], we believe that adequate training is crucial to enable pharmacists to perform these “new public health roles” effectively and safely. Specific training from both a medical and communication perspective would enable pharmacists to become influential figures in their communities: popular, established, and respected figures capable of influencing the behavior and decisions of their audiences [39]. This is especially true in small rural and mountain communities, such as those of the Carnia district we studied, where the pharmacist is often the point of reference for many health issues. For these reasons, we believe that pharmacist involvement is critical to improving adherence to vaccination campaigns. Specifically, by adding to their expertise the personal, cultural, and linguistic knowledge of the area in which they work, an “influencer” pharmacist can increase Confidence in the efficacy and safety of vaccines and in the healthcare system that provides vaccinations, reduce Constraints by increasing the accessibility of the service and improving understanding through the use of easily understandable language, decrease Complacency by educating people about the potential benefits of vaccination, influence the outcome of the Calculation process by providing users with truthful and complete information, and increase the sense of Collective responsibility because of their central role in the community [5]. In Italy in particular, in the new scenario emerging with Legislative Decree 77/2022 and the National Recovery and Resilience Plan (Piano Nazionale di Ripresa e Resilienza, PNRR), which aim to reform the organization of primary care at the national level, the role and competences of pharmacies could soon be integrated with their greater involvement in health promotion and disease prevention activities. Thus, these health professionals could contribute to public health efforts by helping to identify patients with risk factors or unhealthy lifestyles, providing evidence-based education and counseling, countering common misconceptions and fake news, and ultimately empowering the community and individuals to achieve their best health outcomes [40].

The results of this study should be considered in light of several limitations. First, we retrospectively analyzed data collected on individual vaccination intentions, so some patient courtesy bias cannot be ruled out as a matter of respect so as not to offend the pharmacist who provided the counseling. However, to evaluate the effectiveness of the pharmacy-based intervention, we analyzed actual vaccination adherence instead of intention-to-treat data to control for possible misclassifications. Second, we were unable to assess the acceptability of the intervention because no data were collected on interview and counseling refusers. Moreover, the study focused mainly on people aged 65 years or older and those with risk factors, therefore the results could not be generalizable to the general population. Finally, the implementation of this project in the single district of Carnia may have influenced the results due to other particular contextual factors unknown to the researchers, so the considerations made might not be directly transferable to all national or international contexts.

## 5. Conclusions

The pharmacy-based intervention implemented in the mountainous Carnia district of Friuli Venezia Giulia (Italy) proved effective in improving vaccination compliance among the population, particularly among older cohorts. The benefits of this experience were the widespread presence of pharmacies in the area, the recognized and trusted role of pharmacists in the community, and the implementation of specific training for these health professionals to improve the knowledge and skills needed to counsel people on this topic. Considering that these features can be easily imitated and adapted to other public health issues, pharmacies should be more effectively involved in the provision of public health services that seek to improve accessibility, timeliness, and equity. In particular, pharmacists could play a key role in promoting health, for example by promoting initiatives that encourage following a healthy lifestyle (abstention from smoking and alcohol, regular physical activity, balanced diet, etc.) and joining screening programs.

## Figures and Tables

**Table 1:** McNemar test for evaluating vaccination adherence in the 2018–19 and 2019–20 influenza vaccination campaigns for all participants, persons 65 years and older without additional risk factors, and persons 65 years and older with risk factors.

Vaccinated during 2018–2019 Flu Season		Vaccinated during 2019–2020 Flu Season		*p*-Value
		No	Yes	
all participants				
No	766 (37.9%)	175 (22.9%)	591 (77.2%)	*p* < 0.01
Yes	1254 (62.1%)	276 (22.0%)	978 (78.0%)	
>65 years old without additional risk factors				
No	563 (39.6%)	129 (22.9%)	434 (77.1%)	*p* < 0.01
Yes	857 (60.4%)	205 (23.9%)	652 (76.1%)	
>65 years old with other risk factors				
No	203 (33.8%)	46 (22.7%)	157 (77.3%)	*p* < 0.01
Yes	397 (66.2%)	71 (17.9%)	326 (82.1%)	

**Table 2:** Number of persons 65 years and older vaccinated against influenza from 2016 to 2019 at the district, LHA, and provincial levels.

	2016	2017	Δ% 2017–2016	2018	Δ % 2018–2017	2019	Δ % 2019–2018	Chi–Square for Trend
	Vaccinated/ Population (%)	Vaccinated/ Population (%)		Vaccinated/ Population (%)		Vaccinated/ Population (%)		
Carnia	5338/10,265 (52.0%)	5577/10,306 (54.1%)	+4.1	5685/10,703 (53.1%)	−1.8	6487/10,772 (60.2%)	+13.4	<0.01
Gemona	4937/8735 (56.5%)	5029/8783 (57.3%)	+1.3	5188/8879 (58.4%)	+2.0	5537/8966 (61.8%)	+5.7	<0.01
LHA	24,653/44,301 (55.6%)	25,661/44,613 (57.5%)	+3.4	26,266/45,439 (60.2%)	+0.5	28,384/45,874 (63.4%)	+7.0	<0.01
Udine province	75,726/136,540 (55.5%)	78,274/137,555 (56.9%)	+2.6	82,086/139,230 (59.0%)	+3.6	87,256/140,809 (62.0%)	+5.1	<0.01

## Data Availability

All data generated or analyzed during this study are included in this published article.

## Supplementary Materials

The following supporting information can be downloaded at: https://www.mdpi.com/article/10.3390/vaccines12030331/s1, S1: Topics of the training course for pharmacists by the Department of Prevention; S2: Draft for the interview conducted by the pharmacists; S3: Comparison among different settings.
