# Supplementary material for: The Role of Pharmacists in Counteracting Vaccine Hesitancy: Effectiveness of the 2019 Carnia Project in Improving Adherence to Influenza Vaccination among Target Population
Source: Vaccines (Basel). 2024 Mar 20;12(3):331. doi: 10.3390/vaccines12030331 (PMC10974853; doi:10.3390/vaccines12030331)
Supplement: Supplementary file 1 [file vaccines-12-00331-s001.zip › Supplementary material S1_training.pdf]

Supplementary Material S1:

Topics of the training course for pharmacists by the Department of Prevention

Title: “Safety and efficacy of vaccines: Influenza Vaccination Campaign 2019-2020”

Main topics:

- influenza surveillance in Italy
- influenza epidemiology in Italy and in Friuli Venezia Giulia
- virus typing during the 2017-2019 seasons in Italy
- impacts of influenza virus infection on cardiovascular disease and possible protective mechanism of vaccination
- influenza vaccination coverage among the elderly in Friuli Venezia Giulia 1997-2018
- influenza prevention and control: recommendations and targets for the 2019-2020 vaccination campaign
- ministerial guidelines for the definition of risk categories
- available vaccines types: appropriateness, efficacy and safety
- methods for distribution of the vaccine
- instructions for the promotion of influenza vaccination by Carnia district pharmacists
