# Supplementary material for: The Role of Pharmacists in Counteracting Vaccine Hesitancy: Effectiveness of the 2019 Carnia Project in Improving Adherence to Influenza Vaccination among Target Population
Source: Vaccines (Basel). 2024 Mar 20;12(3):331. doi: 10.3390/vaccines12030331 (PMC10974853; doi:10.3390/vaccines12030331)
Supplement: Supplementary file 1 [file vaccines-12-00331-s001.zip › Supplementary material S2_interview.pdf]

Supplementary material S2: draft for the interview conducted by the pharmacists

| | |
|---|---|
| Pharmacy name | |
| Age | |
| Gender | |
| Vaccinated last year? (yes/no) | |
| If "no", why? | □ not getting ill<br>□ vaccines are dangerous<br>□ did not know I could access flu vaccination<br>□ vaccines are not effective<br>□ other |
| Intention to get vaccinated this year (yes/no)? | |
| Presence of risk factors (specify which type) | |
