# Supplementary material for: The Role of Pharmacists in Counteracting Vaccine Hesitancy: Effectiveness of the 2019 Carnia Project in Improving Adherence to Influenza Vaccination among Target Population
Source: Vaccines (Basel). 2024 Mar 20;12(3):331. doi: 10.3390/vaccines12030331 (PMC10974853; doi:10.3390/vaccines12030331)
Supplement: Supplementary file 1 [file vaccines-12-00331-s001.zip › Supplementary material S3_intervention.pdf]

Supplementary material S3: comparison among different settings

	Carnia district	Gemona district	LHA	Udine province
Informative advertising campaign	✔	✔	✔	✔
Vaccinations’ delivery performed by GPs	✔	✔	✔	✔
Vaccines’ distribution	by local pharmacies in several tranches	by the Department of Prevention in a unique tranche		
Training course for pharmacists by the Department of Prevention	✔	x	x	x
Pharmacists’ one-to-one counseling	✔	x	x	x
